# Biomarkers for classification and class prediction of stress in a murine model of chronic subordination stress

**DOI:** 10.1371/journal.pone.0202471

**Published:** 2018-09-05

**Authors:** Dominik Langgartner, Andrea M. Füchsl, Lisa M. Kaiser, Tatjana Meier, Sandra Foertsch, Christian Buske, Stefan O. Reber, Medhanie A. Mulaw

**Affiliations:** 1 Laboratory for Molecular Psychosomatics, Clinic for Psychosomatic Medicine and Psychotherapy, Ulm University, Ulm, Germany; 2 Institute for Experimental Cancer Research, Comprehensive Cancer Center Ulm, Ulm University, Ulm, Germany; 3 Clinic for Psychosomatic Medicine and Psychotherapy, Ulm University, Ulm, Germany; University of California Los Angeles, UNITED STATES

## Abstract

Selye defined stress as the nonspecific response of the body to any demand and thus an inherent element of all diseases. He reported that rats show adrenal hypertrophy, thymicolymphatic atrophy, and gastrointestinal ulceration, referred to as the stress triad, upon repeated exposure to nocuous agents. However, Selye’s stress triad as well as its extended version including reduced body weight gain, increased plasma glucocorticoid (GC) concentrations, and GC resistance of target cells do not represent reliable discriminatory biomarkers for chronic stress. To address this, we collected multivariate biological data from male mice exposed either to the preclinically validated chronic subordinate colony housing (CSC) paradigm or to single-housed control (SHC) condition. We then used principal component analysis (PCA), top scoring pairs (tsp) and support vector machines (SVM) analyses to identify markers that discriminate between chronically stressed and non-stressed mice. PCA segregated stressed and non-stressed mice, with high loading for some of Selye’s stress triad parameters. The tsp analysis, a simple and highly interpretable statistical approach, identified left adrenal weight and relative thymus weight as the pair with the highest discrimination score and prediction accuracy validated by a blinded dataset (92% p-value < 0.0001; SVM model = 83% accuracy and p-value < 0.0001). This finding clearly shows that simultaneous consideration of these two parameters can be used as a reliable biomarker of chronic stress status. Furthermore, our analysis highlights that the tsp approach is a very powerful method whose application extends beyond what has previously been reported.

## Introduction

Hans Selye defined stress as the nonspecific response of the body to any demand and is an inherent element of all diseases [1,2]. The typical stress triad in rats after severe damage by diverse nocuous agents consists of adrenal hypertrophy and thymicolymphatic atrophy (a decrease in thymus, lymph nodes, and spleen weight), as well as gastrointestinal ulceration [1,2]. Stressors used by Selye include exposure to cold, surgical injury, production of spinal shock, excessive muscular exercise, intoxications with sub-lethal doses of diverse drugs (adrenaline, atropine, morphine, formaldehyde, etc.) [2]. Since these pioneering reports, an enormous number of human and animal studies on chronic stress has been published. They largely agreed on decreased body weight gain [3–7], hyper-activation of the hypothalamus-pituitary-adrenal (HPA) axis and, thus, increased basal plasma glucocorticoid (GC) and adrenocorticotropic hormone (ACTH) concentrations [8–11], as well as GC resistance of target tissues [8,12–18] to represent additional specific and species-independent chronic stress features.

However, own data and data from others clearly show that the typical stress triad, or the extended version of it, actually do not provide adequate discrimination power between chronically stressed and non-stressed individuals and, thus, are not reliable biomarkers of chronic stress. For instance, many studies done in either humans or animals report also unaffected or decreased plasma GC and ACTH levels [17,19–28], unaffected or increased spleen [14,29], lymph node [30], and body weight [31–35], and no signs of gastrointestinal ulceration [19,29,36] or GC resistance of target cells [15,16,37]. To our knowledge, only increased adrenal weight and decreased thymus weight are consistently reported following chronic stressor exposure in animals. Though to our knowledge the latter parameters have not been assessed in many human stress studies so far, increased adrenal weight was found in depressed versus non-depressed patients using computed tomography [38] and in suicide victims versus sudden death, non-psychiatric controls using post-mortem analysis [39].

In the current study, we first measured various physiological and immunological parameters in male mice exposed to either the chronic subordinated colony housing (CSC) paradigm, a preclinically validated model of chronic stress [17,29,40–42], or single-housed control (SHC) condition [43]. In the second step, we employed a simple and highly interpretable classification approach to delineate chronically stressed and non-stressed mice. The method, top scoring pair (tsp), identifies pair/s of parameters that show the maximum difference in ranking between two groups. To our knowledge, tsp has mainly been used in high throughput expression studies and we are the first to show here that its application extends beyond those data types. Furthermore, due to the non-parametric nature of the approach and that it relies only on relative rank of a pair of variables [44], it has an advantage over methods that depend on normal distribution including various regression models.

## Material and methods

### Animals

Male C57BL/6 mice (Charles River, Sulzfeld, Germany) weighing 19–22 g were individually housed in standard polycarbonate mouse cages (16 x 22 x 14 cm) for one week before starting the CSC paradigm. Male CD-1 mice (Charles River, Sulzfeld, Germany; weighing 30–35 g) were used as dominant residents. All mice were kept under standard laboratory conditions (12 h light/ dark cycle, lights on at 0600 h, 22°C, 60% humidity) and had free access to tap water and standard mouse diet. All experimental protocols were approved by the Committee on Animal Health and Care of the local government (IACUC, Regierungspräsidium Tübingen, Baden-Württemberg, Germany, permit numbers AZ 35–9185.81–3: 1136, 1216), and have been conducted according to applicable national and international guidelines on the ethical use of animals. All efforts were made to minimize the number of animals used and their suffering. Some of the animals included in the statistical analysis of the present study were included in a recently published study [16,45–47].

### Experimental procedures

Chronic stress was induced by the pre-clinically validated chronic subordinate colony housing (CSC) paradigm [17,29,40–42]. To maximize statistical power, data homogeneity, and minimize the number of experimental animals, we used all CSC experiments performed independently by our group during the last two years. All experimental mice were exposed to 19 days of either CSC or SHC and killed on day 20 by rapid decapitation following brief CO_2_ exposure.

### Chronic subordinate colony housing (CSC)

CSC paradigm was conducted as described previously [42,43,48–50]. Briefly, four experimental CSC mice were housed together with a dominant male mouse for 19 consecutive days to induce a chronic stressful situation. Before the CSC procedure, the future dominant males were tested for their aggressive behavior. Males that started to injure their opponents by harmful bites were not used. To avoid habituation, each dominant male was replaced by a novel dominant male at days 8 and 15 of the CSC procedure. SHC mice remained undisturbed in their home cages except for a change of bedding once a week. In a previous study, we convincingly demonstrated that single housing is the adequate control group for the CSC paradigm, as group housing itself was shown to be stressful and affects parameters assessed routinely in studies employing the CSC paradigm [43].

### Parameters

#### Body weight gain

SHC and CSC mice were weighed on day 1 before the start of the CSC paradigm and on day 20 before decapitation. Body weight gain was calculated according to the following formula: body weight on day 20—body weight on day 1.

#### Trunk blood sampling

On day 20, all SHC and CSC mice were rapidly euthanized by decapitation following brief CO_2_ exposure between 0700 and 1000 h. Blood drawing for each mouse was finished within 3 min after entering the animal room. Trunk blood was collected in EDTA-coated tubes (Sarstedt, Nümbrecht, Germany) on ice and centrifuged at 4°C (5000 g, 10 min). Plasma samples were stored at– 20°C until assayed.

#### ELISA for CORT

Plasma samples were analyzed using commercially available ELISA for CORT (Analytical sensitivity < 1.631 nmol/L, intra-assay and inter-assay coefficients of variation ≤ 6.35%, IBL International, Hamburg, Germany).

#### Determination of adrenal, pituitary, spleen, and thymus weight

After decapitation on day 20, the left and right adrenal glands, the pituitary, the spleen, and the thymus of each animal were removed, pruned of fat, and weighed separately.

#### Characterization of GC sensitivity of isolated splenocytes

In order to determine the effects of CSC on GC dependent cell viability of splenic cells, splenocytes were isolated and adjusted to a final cell concentration of 5 x 10^6^ cells/ml as described previously [17]. Cell suspensions were then stimulated with lipopolysaccharide (LPS; final concentration was 1 μg/ml) from *Escherichia coli* (serotype O111:B4, Sigma-Aldrich, St. Louis, MO) or remained untreated to assess background activity. To determine the GC sensitivity of unstimulated and LPS-stimulated cells, aliquots of each sample were treated with various concentrations of CORT (Sigma-Aldrich; final concentrations were 0.005, 0.05, 0.1, 0.5, and 5 μM, respectively). We used both physiological and pharmacological doses of the hormone and cells were incubated for 48 h in a humidified incubator (37 °C, 5% CO_2_) followed by an assessment of cell viability. The cell viability in the splenocyte cultures was determined with a commercially available colorimetric assay (CellTiter 96 AQueous One Solution Assay, Promega G3580).

#### Isolation of mesenteric lymph node cells

To assess CSC effects on the number of viable mesenteric lymph node cells, mesenteric lymph nodes were removed and stored in ice-cold Roswell Park Memorial Institute Medium (RPMI-1640; Sigma-Aldrich Corp., St. Louis, MO, USA) until all animals were euthanized. Mesenteric lymph node cells were isolated and stimulated as described previously [17]. Briefly, mesenteric lymph nodes (pooled from each experimental group) were harvested under sterile conditions and collected on ice in cell culture medium [RPMI-1640 supplemented with 10% fetal calf serum (Biochrom, Germany), 100 U/ml penicillin and 100 μg/ml streptomycin (GIBCO-BRL, Eggenstein, Germany) and 3 x 10^−5^ M ß-mercaptoethanol (Sigma, Deisenhofen, Germany)]. Lymph nodes were mechanically disrupted and filtered through a cell strainer (70 *μ*m Nylon, FalconTM, Becton Dickinson, Germany). Afterwards, cells were washed three times in cell culture medium and counted. Non-viable cells were visualized using trypan blue.

### Statistical analysis

All statistical analyses were performed in R using published packages and in-house scripts.

#### Principal component analysis (PCA)

Principal component analysis was performed using the package prcomp [51–53]. The three dimensional plot and biplot with variable loadings were generated using the top three principal components.

#### Prediction models

Top scoring pairs (tsp) analysis was done using the tspair package [44]. Fifty-seven SHC (non-stressed) and CSC (stressed) mice were used to train the model. The method takes two variables at a time and assigns a score. Pairs with the highest score were considered for subsequent analysis. The model was further validated using 37 independent blinded set of stressed and non-stressed mice. Predicted status obtained from the model was compared to the actual status to calculate the empirical prediction error (EPE).

Support vector machines (SVM) analysis was done using the kernlab package [54]. The pair of variables with the highest score (left adrenal weight [mg] and relative thymus weight [mg/g]) and the 57 mice used in the tsp modeling were also used to train the SVM model. We used the support vector classification parameter (C-svc) function with Laplacian kernel. Similar to the tsp model, we used the blinded dataset of 37 stressed and non-stressed mice to validate the model. EPE was calculated by comparing SVM predicted stress status and empirical annotation of the validation set.

#### Monte Carlo Simulation and p-values

To obtain the null hypothesis distribution of prediction accuracy of the models, we first generated random values in the range of the left adrenal weight [mg] and relative thymus weight [mg/g] of the 37 stressed and non-stressed mice. We then conducted 10000 iterations by sampling these values and obtaining the prediction error for each loop. Mean prediction error was obtained by taking the average of the 10000 prediction error values. These values were later plotted as dot plot and kernel density function plot to simplify visualization. P-values of the EPE of both models were obtained by using the formula *P* = *r* + 1/*N* + 1 where *r* is the number of observations in the null distribution that had lower prediction error than the EPE of the model and *N* is the total number of iterations.

#### Bootstrap and confidence interval analysis

We wrote an R function that takes the model of interest and the validation data set as input and returns the encountered prediction error for a given subset of the data. The R boot package [55,56] was then used to perform bootstrap analysis with 10000 replicates on this function and the prediction error of each round of iteration, which was the statistic of interest for further analysis. The 95% percent confidence interval was obtained for each model using the adjusted bootstrap percentile (BCa) method. The mean prediction error and the 95% CI of the bootstrap analysis were then plotted using dot plot and kernel density function.

## Results

### Stressed and non-stressed mice segregate into distinct clusters based on principal component analysis

**Fig 1:**
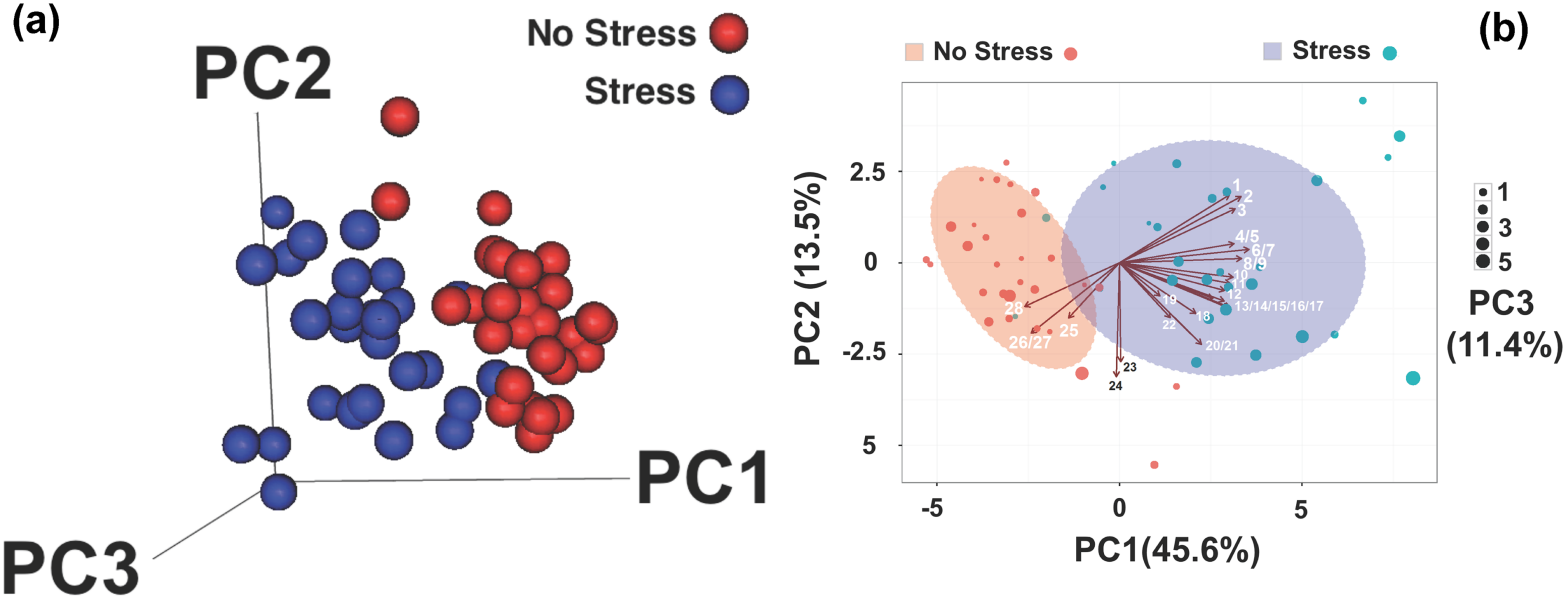
(a) A 3D plot showing the result of the Principal component analysis (PCA) using all the 28 physiological and immunological parameters of non-stressed (SHC or No Stress; red) and stressed mice (CSC or Stress). The first three components shown here cumulatively explain 70.5% of the variation in the dataset, segregating the SHC and CSC mice. (b) A bi-plot showing the PCA analysis results (as shown in Fig 1a) with additional information on loadings for each variable in the analysis. The direction and length of arrows indicate the sign and magnitude of the coefficient of each variable in the PC1 and PC2 coordinate, respectively. Size of the balls indicates PC3 coefficient loadings in scales 1 to 5 (shown the right side of the plot). Light red shades and red balls represent SHC samples while purple shades and light blue balls indicate CSC mice. The corresponding amount of variation explained by each component is given in brackets. Details of the numeric labels for each arrow are given in S3 Table.

We performed principal component analysis (PCA) based on the different parameters measured in both CSC (stressed) and SHC (non-stressed) mice (see Methods for the list of parameters and S1 Table). To account for differences in scale and variability of parameters, the PCA analysis was performed on centered and scaled values. The first seven components explained more than 90% of the variation (S2 Table). We then looked at the top three principal components and noted that the stressed and non-stressed mice segregate into two groups (Fig 1a and 1b; cumulative explained variation of 70%). We observed relatively higher heterogeneity in the stressed mice as compared to the non-stressed ones. Additionally, we noted that the different parameters in the study showed varying degree of association with the stress status (Fig 1b). Accordingly, higher adrenal weight measurements (left and right adrenal weight and relative adrenal weight; arrows numbered 1 to 9 in Fig 1b) were positively associated with stressed mice while higher thymus weight (both absolute and relative; arrows 26–28 in Fig 1b) were associated with non-stressed mice (complete annotation of arrows is given in supplementary S3 Table). Measurements like body weight (both absolute and gained weight) showed little or no association with either group (arrow numbers 23 and 24 in Fig 1b).

### Chronic stress status can be predicted by a bivariate left adrenal weight and relative thymus weight signature

**Fig 2:**
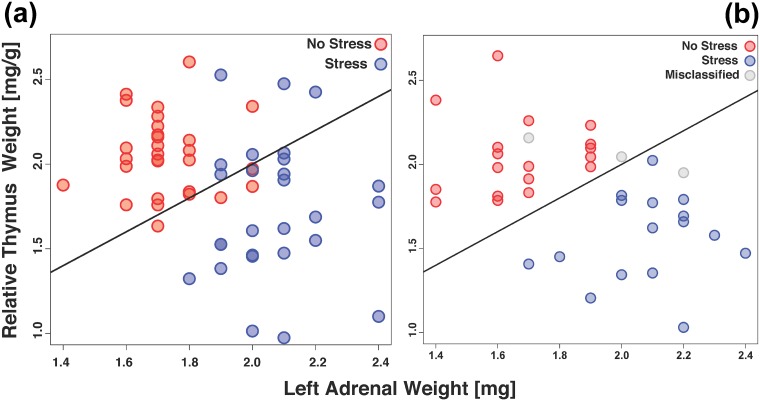
(a) Scatter plot based on top scoring pairs (tsp) analysis of the training dataset aimed at classifying stressed (red) and non-stressed (blue) samples. The Left Adrenal Weight [mg] (LAWmg; x-axis) and Relative Thymus Weight [mg/g] (RTWmg/g; y-axis) were identified as the most relevant pair. The fitted line represents the linear function that discriminates the two groups. (b) The model was tested using independent validation set of 37 samples. The obtained results are shown here as a scatter plot. Correctly predicted samples (34/37 or 91.89%) are colored by matching to the colors of the test set (red = No Stress; blue = Stress). Misclassified samples (n = 3) are shown in grey.

Following our observation of the PCA, we set out to develop a stress signature that aims at classifying samples into stressed and non-stressed groups based on the measured parameters. Furthermore, the model should predict the stress status of samples with high accuracy and statistical significance. To achieve this, we first employed the top scoring pair (tsp) approach where all pairwise combinations of the variables in a training set are evaluated (see Methods for details). The tsp analysis showed that the left adrenal weight [mg] (LAWmg) and relative thymus weight [mg/g] (RTWmg/g) gave the highest classification score based on a training set of 57 stressed and non-stressed mice (Fig 2a). We observed here that the stressed mice showed higher LAWmg and lower RTWmg/g in contrast to the non-stressed mice, which goes in line with our PCA findings. To assess the class prediction error of the model, we used a validation set of stressed and non-stressed mice (n = 37) and observed that the model was able to correctly predict the stress status of 34 individuals (~92% accuracy or ~8% prediction error) (Fig 2b, Tables 1 and 2 and S4 Table). Of these, one SHC (No Stress) and two CSC (Stress) mice were misclassified (Table 1).

**Table 1:** Class prediction accuracy of the top scoring pair (tsp) model (left adrenal weight [mg] and relative thymus weight [mg/g]). Only one mouse in the SHC (No stress; n = 19) and two individuals in the CSC (Stress; n = 18) were misclassified.

		**Predicted status**		
		No Stress	Stress	
**Actual status**	No Stress	18	1	19
	Stress	2	16	18
		20	17	**37**

**Table 2:** Tsp model prediction accuracy. The model correctly predicted the stress status of 34 mice out of 37 mice in the validation set (~92%).

Classification accuracy	Count	Percentage
**Misclassified**	3 (2+1)	8.11%
**Correctly classified**	34 (18+16)	91.89%
**Total**	37 (3+34)	100.00%

**Fig 3:**
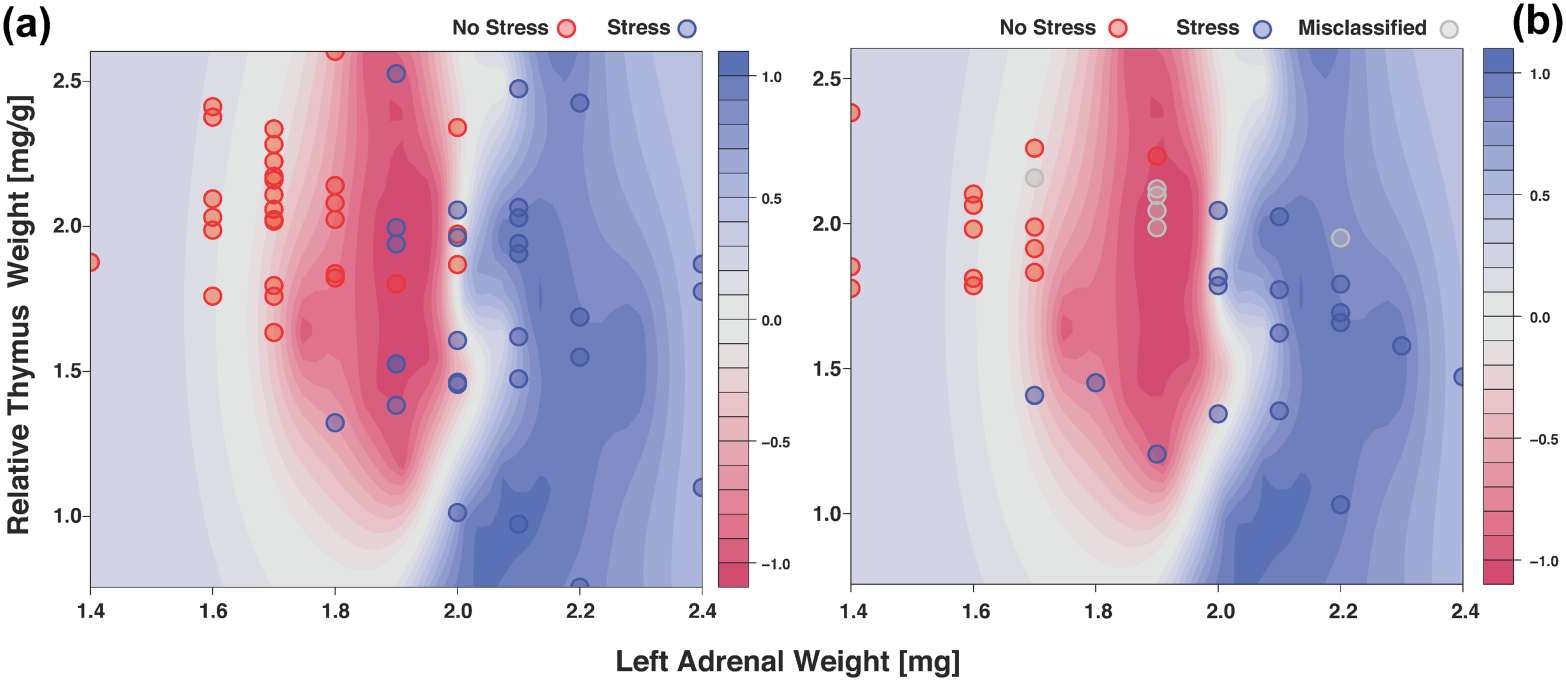
(a) Contour plot depicting Support vector machines (SVM) analysis results using the LAWmg (x-axis) and RTWmg/g (y-axis) parameters (see Methods for details of parameter settings used for analysis). Red contours represent non-stressed sample boundaries while blue covers the stressed samples. Points represent samples. (b) Correctly predicted samples (31/37 or 83.78%) are shown in this plot and are colored by matching to the colors of the test set (red = No Stress; blue = Stress). Misclassified samples (n = 6) are colored grey.

To assess if other prediction approaches yield similar results, we used LAWmg and RTWmg/g as input in an SVM analysis. In line with our tsp analysis, SVM analysis based on LAWmg and RTWmg/g showed low training error (0.035) and low internal cross-validation error (0.088) (Fig 3a). We then used the mice in our validation set (n = 37) to test the class prediction accuracy of the model. We also saw here that the model performed well in predicting stress status (31/37 or ~84%; Fig 3b, Tables 3 and 4), albeit slightly lower accuracy as compared to the tsp model. Five of the non-stressed mice were misclassified as CSC (Stress) while one stressed mouse was identified as SHC (No Stress).

**Table 3:** Table showing the stress status prediction based on support vector machine (SVM). Five mice in the SHC (No Stress) and 1 in the CSC (Stress) were incorrectly predicted.

		**Predicted status**		
		No Stress	Stress	
**Actual status**	No Stress	14	5	19
	Stress	1	17	18
		15	22	**37**

**Table 4:** SVM model prediction accuracy. Of the 37 mice in the validation set, the model correctly assigned 31 individuals to their appropriate groups (~84%).

Classification accuracy	Count	Percentage
**Misclassified**	6 (1+5)	16.22%
**Correctly classified**	31 (14+17)	83.78%
**Total**	37 (6+31)	100.00%

**Fig 4:**
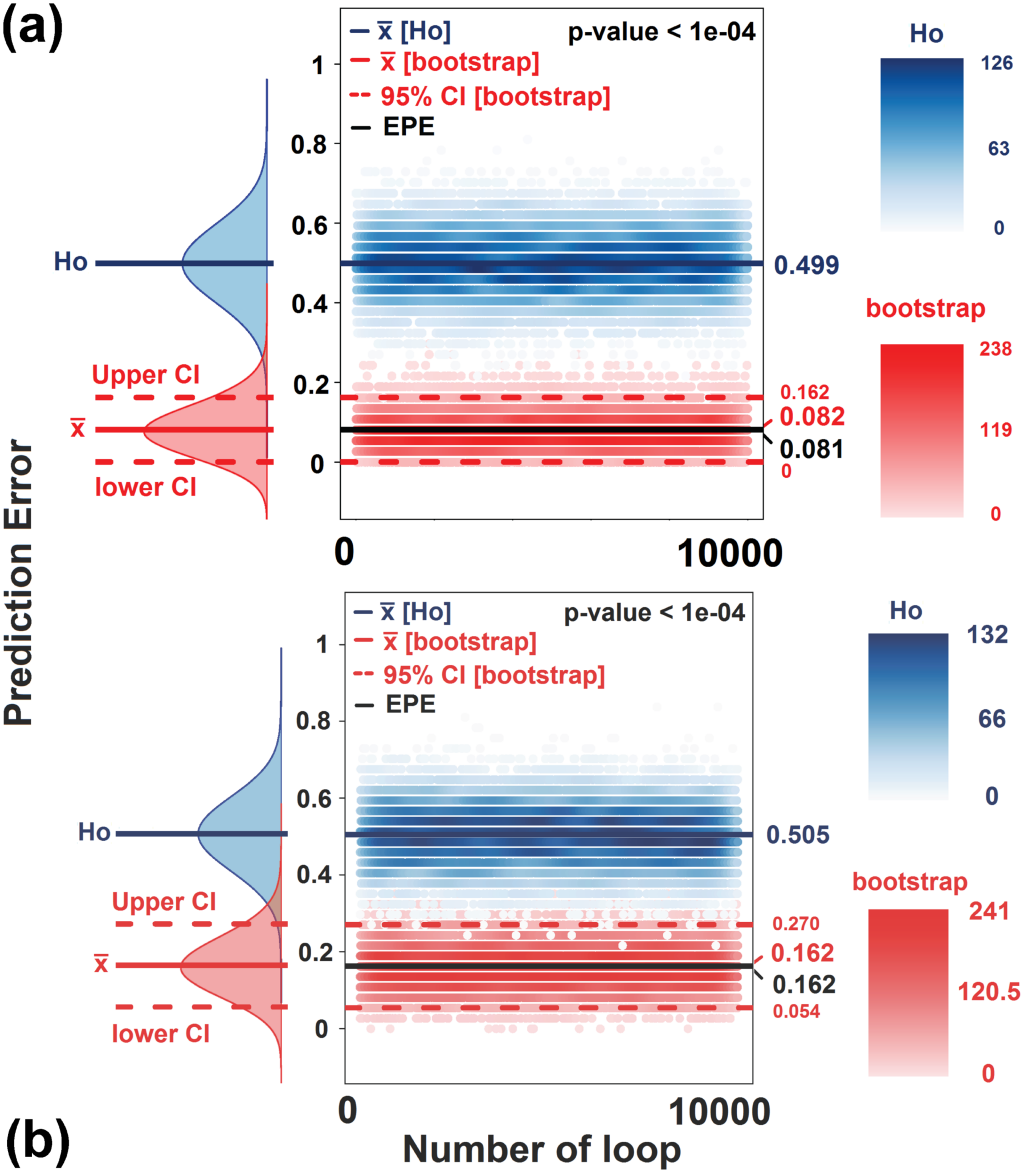
(a) A plot showing null hypothesis distribution generated using Monte Carlo Simulation (blue density and scatter plots) and bootstrap based mean and confidence interval estimation of the prediction error encountered by the tsp model (red density and scatter plots). The x-axis shows the number of iterations/replicates while the y-axis shows the prediction error. Dark blue lines, both in the density plots and scatter plots represents estimated mean of the null hypothesis ($\overset{-}{\text{x}}$ [Ho]). Full red line is the mean prediction error ($\overset{-}{\text{x}}$ [boostrap]) as estimated by the bootstrap analysis while the dashed red lines are the corresponding 95% Confidence Intervals (95% CI [bootstrap]). The black line indicates the empirical prediction error (EPE) obtained in the validation set analysis. (b) A Plot of the statistical significance and confidence interval of the prediction analysis of the SVM model (details of the labels are as described in Fig 4a).

To estimate the statistical significance of the prediction accuracy, we first generated a null hypothesis distribution of misclassification error for both models by employing Monte Carlo Simulation (see Methods for details). Mean misclassification errors of the null hypotheses were 0.499 and 0.505 for the tsp and SVM models, respectively (see Fig 4a and 4b). This fits the expected rate of a random class prediction, where there is an equal likelihood of being placed into either the stressed or the non-stressed group. We then estimated the p-value of the prediction error of the models against the null hypothesis distribution and both were statistically significant (p values < 0.0001; Fig 4a and 4b). We finally calculated mean prediction errors and confidence intervals of the models using non-parametric bootstrap analysis. The mean prediction error for the tsp model was 0.082 with upper and lower 95% confidence intervals (CI) of 0.16 and ~0, respectively. The bootstrap mean prediction error of the tsp model was in line with the actual prediction error obtained in the validation set (0.081) (Fig 4a). Similarly, the mean prediction error of the SVM model was 0.162 with an upper 95% CI of 0.270 and lower 95% CI of 0.054. In line with the observation in the previous model, bootstrap mean prediction error of the SVM model was equivalent to the empirical prediction error (Fig 4b).

Taken together, these results clearly show that simultaneous consideration of the left adrenal weight and relative thymus weight significantly predict the psychosocial stress status of our mouse model and sufficiently capture the physiological response to stress without the need for additional stress markers.

## Discussion

Employing state of the art statistical analysis, we are able to show in the current study that an increase in absolute left adrenal weight and a decrease in relative thymus weight are the most reliable stress biomarkers in a mouse model of chronic subordination stress, allowing the best possible recapitulation of the chronic stress status for each individual mouse. Although largely in line with the stress triad proposed by H. Selye [57], we could not confirm shrinking of peripheral lymph nodes and the spleen as well as signs of gastrointestinal inflammation to represent reliable and stressor independent biomarkers of chronic stress. Selye stated that it is difficult to label an effect as a stress response until the same effect is reproduced by several stressors different in nature [57], i.e. changes in these latter parameters should be interpreted with caution, as they might reflect specific rather than non-specific consequences of certain stress paradigms or challenges. The aim of the current study is thus not to statistically prove that all reported consequences of CSC exposure represent common features of chronic stress in general or chronic psychosocial stress in particular. One would require, as afore-mentioned, a number of stressors to make such generalized statements [58]. We rather sought to use the CSC paradigm as one more model of chronic stress, in addition to the ones employed by Selye and others, to provide statistical evidence that not the stress triad or the extended stress triad, but a pair of reliable discriminators might be sufficient to classify stressed and non-stressed individuals.

To address this point and statistically recapitulate Selye’s stress triad, or the later developing extended version of it, we assessed various physiological and immunological parameters from mice that have been exposed to either the CSC [41] paradigm or to SHC conditions. We first analyzed the data using PCA considering all data points and variables (S1 Table). We then employed a top scoring pair (tsp) analysis to identify parameters that could discriminate between CSC and SHC mice. The method has been widely used in gene expression profiling-based classification and prediction analysis [59–62] and has gained popularity in various types of -omics studies [63–66]. We show here for the first time that the application extends beyond such datasets. The simple and highly interpretable approach is based on the identification of pair/s of parameters that show the maximum difference in ranking between two groups.

We identified absolute left adrenal weight (mg) and relative thymus weight (mg/g) as the pair with the highest discrimination score and prediction accuracy of stress status (approx. 92%; Figs 2 and 3 and Tables 1 to 4). This is not just in line with Selye’s stress triad but also with many published studies [4,7,8,11,14,17,20,21,29,31,48,67–69]. The better predictive power of the left versus right adrenal weight in the current study might simply be due to the fact that the left adrenal is larger than the right adrenal in a species-independent manner [20,70–72]. This possibly reflects a generally increased sensitivity to the trophic factor ACTH, which is also released during chronic stress. However, as there are studies showing that chronic voluntary wheel running in rats, reflecting a physical rather than psychosocial stressor, specifically increases the right but not left adrenal weight [71–73], body side specific effects of chronic stress on adrenal weight might also be related to the type of chronic stressor faced. Further in line with our findings, increased adrenal weight in suicide victims compared with sudden death, non-psychiatric controls was shown to be mediated by the left adrenal gland [39]. Future studies are warranted to address this in detail.

Our current work and the studies mentioned above faithfully recapitulate the original landmark observations made by Selye regarding thymus and adrenals as readily responding organs when an organism gets exposed to various types of stressors [74] but do not support the development of gastrointestinal inflammation as a general consequence of chronic stress. These data are in line with results of a recent study from our group, showing that the vulnerability to develop CSC-induced colitis is strongly dependent on the presence of certain intestinal pathobiontic germs [75], as for instance certain enterohepatic *Helicobacter* spp. [47,76]. In detail, in contrast to non-specific pathogen free (non-SPF) conditions, under which CSC exposure reliably causes spontaneous colitis [17,77], CSC animals did not show any signs of intestinal inflammation when performing the paradigm under SPF conditions. Importantly, reintroducing *Helicobacter typhlonius* into otherwise SPF conditions was able to rescue the colitogenic potential of CSC [47], clearly indicating that stress-induced intestinal inflammation is not representing a nonspecific and stressor independent general consequence of chronic stress in the sense of Selye’s stress triad but rather depends on whether certain pathobionts are present or not during stressor exposure. The same is true for the stress-induced shrinkage of peripheral lymph nodes, initially proposed by Selye as part of the stress triad. The number of viable mesenteric lymph node cells was comparable between CSC and SHC animals, independent of the presence of certain intestinal pathobionts [47]. Further in contrast to the stress triad proposed by Selye, statistical analysis performed in the current study did not support a decrease in spleen weight to be a reliable biomarker of chronic stress. If anything, our data suggest CSC exposure to promote an increase instead of a decrease in spleen mass, in line with our previous findings, showing increased spleen weight in CD1 mice exposed to 19 days of CSC [29]. Of note in this context, studies using another chronic stress paradigm, namely the social disruption (SDR) model, also describe splenomegaly as a consequence of disrupting the hierarchical structure in mice by daily introducing an aggressive conspecific over 6 consecutive days [14,78].

Parameters added later to the extended version of the stress triad by Selye or other stress researchers were a decreased body weight gain and increased plasma GC concentrations [1,9]. However, statistical analysis in the current study did not delineate a decreased body weight gain or increased basal plasma CORT concentrations as reliable indicators for CSC exposure in particular and for chronic stressor exposure in general. Although an increased adrenal weight and increased basal plasma ACTH levels on Day 20 of CSC [42] clearly indicate continuous HPA axis activation during CSC exposure, plasma corticosterone levels are elevated only during the first 10–24 hours [20,79], before they normalize again, probably representing an adaptation during continuous stressor exposure. This is also in line with own previous studies, showing either a decreased or an unaffected body weight and unaffected (morning) or lower (evening) plasma CORT concentrations following 19 days of CSC exposure. Similar observations have also been reported by others [42].

The current study has several strengths but also some limitations that warrant consideration. Notable strengths are the use of a standardized, well-characterized and highly reproducible chronic stress paradigm as well as the combination of both *in vivo* and *ex vivo* techniques to assess possible stress consequences. In addition, the classification and class prediction approach we have employed has an advantage over other methods that depend on normal distribution as it is non-parametric by nature and relies only on relative rank of a pair of variables [44,63]. Furthermore, the model is simple as it solely is based on a single function of *y* = *x* to define class boundaries. This has the added advantage as discrimination score of pairs of parameters can easily be compared and ranked.

The study has some limitations. We have to mention that the CSC paradigm allows physical contact between the male conspecifics and, therefore, is not exclusively psychosocial in nature, making it hard to conclude whether an increased absolute left adrenal and a decreased relative thymus weight represent reliable biomarkers of chronic stress in general or of chronic psychosocial stress in particular. Additionally, as there might be a species-specific stress response, it is likely that comparisons across species might have some drawbacks. Further limitation of the study is the use of only male experimental mice. Given that females are more likely to develop stress-associated mood disorders as compared to males [80], future studies need to address sex differences in terms of chronic stress biomarkers. Finally, the tsp analysis may not always yield a pair with statistically significant classification and class prediction power. In such instances, as mentioned before, one can consider using tsp for feature selection [81] and use approaches that provide more flexibility in terms of model selection and parameter settings.

We believe that our findings are of significance as, first, we show that relative thymus weight and left adrenal weight are the parameters with the highest contribution in the principal component analysis that showed clear segregation of stressed and non-stressed mice. Secondly, we were able to show that relative thymus weight and left adrenal weight could classify stressed and non-stressed mice with high statistical significance and confidence using state of the art analytic approaches (tsp and SVM). Third, by going beyond the dataset used to generate the classification model, we employed a large blinded dataset and could show that we were able to predict the “Stress-status” of each individual mouse with significant accuracy (Fig 4 and Tables 1 to 4). In contrast, a decrease in spleen weight and peripheral lymph node mass, development of gastrointestinal alterations, a decrease in body weight gain and increased plasma CORT concentrations seem to be stressor-specific rather than stressor non-specific responses and do not qualify as adequate stress biomarkers. Therefore, our results argue for the sufficiency of absolute left adrenal and relative thymus weight to recapitulate stress condition, thereby minimizing the number of parameters required and simplify data analysis procedures. Finally, the relevance of new biological and social parameters as indicators of stress conditions could simply be determined by looking at the significance of correlation that they show to both left adrenal weight and relative thymus weight. This clearly shows that simultaneous consideration of relative thymus weight and left adrenal weight are not only associated with stress but are also significant class predictors (stressed vs non-stressed individuals), thereby serving as powerful markers of stress status in psychosocial chronic stress models. Although our conclusions are solely based on findings in male mice and one additionally needs to take into account species-specific stress response, they strongly argue for large-scale screening studies assessing noninvasively the weight/size of these organs by computed tomography, with the aim to find and characterize novel and reliable human biomarkers for chronic stress.

## Supporting information

S1 Table Data matrix showing parameters measured in the current study. Measurements (and their units) are indicated.
(XLS)

S2 Table Table showing the results of principal component analysis (PCA). Standard deviation, the proportion of variance explained, and cumulative proportion across all components are shown.
(XLS)

S3 Table Numbers corresponding to the labels shown in Fig 1b. Colors match the direction of arrows as shown in Fig 1b, blue indicating parameters with positive loading for "Stress" group while red indicates "No Stress" group association. Parameters that are not colored show no association to either group.
(XLS)

S4 Table Predicted group membership of tsp and SVM models in the blinded dataset. Actual status is also shown.
(XLS)
